# Appliance‐Supported Decompression for Radicular Cysts Associated With Primary Molars: A Pediatric Case Series

**DOI:** 10.1155/crid/8619647

**Published:** 2026-09-28

**Authors:** Erenay Alpayçetin, Merve Mısır, Elif Bahar Tuna İnce, Mine Koruyucu, Koray Gençay

**Affiliations:** ^1^ Department of Pediatric Dentistry, Faculty of Dentistry, Istanbul University, Istanbul, Türkiye, istanbul.edu.tr

**Keywords:** decompression, pediatric dentistry, primary teeth, radicular cyst

## Abstract

**Introduction:**

Radicular cysts associated with primary teeth are uncommon in pediatric patients but may cause extensive bone destruction and displacement of developing permanent tooth germs. Conventional surgical enucleation may increase the risk of damage to adjacent structures; therefore, conservative treatment approaches such as marsupialization and decompression are preferred. Appliance‐supported decompression systems have been increasingly used to maintain continuous drainage while preserving the eruption potential of permanent teeth.

**Case Series:**

This case series presents three pediatric patients aged 7–8 years diagnosed with radicular cysts, the most common type of radicular cyst, associated with primary mandibular molars. Following extraction of the affected primary teeth, conservative management was performed using decompression supported by fixed or removable appliances. Radiographic evaluation revealed large radiolucent lesions with varying degrees of displacement of permanent successors. Appliance design was individualized according to lesion characteristics and patient cooperation. Progressive reduction in cyst size, satisfactory bone regeneration, and spontaneous eruption or favorable repositioning of permanent teeth were observed during follow‐up periods ranging from 3 to 20 months. No recurrence or major complications were detected.

**Conclusion:**

Appliance‐supported decompression is a minimally invasive and effective treatment option for inflammatory radicular cysts associated with primary teeth. This approach promotes bone regeneration, preserves permanent tooth eruption potential, and reduces the need for aggressive surgical interventions. Early diagnosis and regular follow‐up are essential for achieving favorable clinical outcomes in pediatric patients.

## 1. Introduction

Radicular cysts are inflammatory odontogenic cysts originating from the epithelial remnants of the periodontal ligament, known as the cell rests of Malassez, as a consequence of pulp necrosis and periradicular inflammation [1, 2]. They represent the most common cysts of the jaws, accounting for approximately 7%–54% of all odontogenic cysts in the permanent dentition [1, 3]. In contrast, radicular cysts associated with the primary dentition are extremely rare, with a reported prevalence ranging from 0.5% to 3.3%, largely due to the physiological resorption and exfoliation of primary teeth [4, 5].

The majority of radicular cysts in children develop secondary to apical infection caused by dental caries, which is considered the most significant etiological factor [2, 4]. Pulp inflammation and subsequent necrosis stimulate inflammatory cell infiltration, including leukocytes and monocytes, leading to proliferation of the epithelial rests and cyst formation [3]. Although pulp therapy is the treatment of choice for primary teeth affected by pulpitis or pulpal necrosis, cyst development has been reported as a potential complication [5, 6]. Antigenic and necrotic by‐products generated by pulp therapeutic agents may provoke an antigenic stimulus in the periradicular tissues, thereby contributing to cystogenesis [4]. Additionally, traumatic injury to primary teeth has also been implicated as a possible etiological factor [6].

Radicular cysts of primary teeth are often asymptomatic and are frequently detected incidentally during routine radiographic examinations [4, 7]. Radiographically, they typically present as well‐circumscribed, round or oval periapical radiolucent lesions, usually surrounded by a radiopaque margin, although this margin may be indistinct or absent in infected or rapidly expanding cysts [1, 2]. If left untreated, radicular cysts may enlarge and result in bone expansion, resorption, tooth mobility, delayed exfoliation of the primary tooth, displacement or eruption disturbances of the succedaneous permanent tooth, and developmental defects of the permanent dentition [6, 8, 9].

Management strategies vary depending on factors such as the patient′s age, lesion size, anatomical location, and the eruption potential of the associated teeth [9]. While enucleation remains the standard treatment for smaller lesions, this procedure carries a higher risk of damaging developing tooth germs and surrounding structures in pediatric patients [8]. Consequently, more conservative approaches are preferred in children [9, 10]. Marsupialization and decompression techniques reduce intracystic pressure and promote gradual lesion shrinkage, thereby minimizing surgical morbidity and facilitating the spontaneous eruption of the involved teeth [10, 11]. For large cystic lesions, a two‐stage treatment approach consisting of initial marsupialization or decompression followed by enucleation has been reported with favorable outcomes [11, 12]. Conservative approaches are also better tolerated by children and their parents and are associated with higher rates of successful preservation of permanent teeth in the mixed dentition period [9, 10].

In recent years, various fixed and removable appliances have been developed to enhance the effectiveness of marsupialization. These appliances not only maintain communication between the cystic cavity and the oral environment to ensure long‐term decompression but also support minimally invasive management of large lesions. Additionally, they can function as space maintainers, preserving the area until the permanent tooth erupts [13].

Although the terms marsupialization and decompression are frequently used interchangeably in the literature, they represent distinct conservative techniques. Marsupialization refers to the surgical exteriorization of the cyst, in which the margins of the cystic lining are sutured directly to the adjacent oral mucosa, creating a permanent surgical window that allows the cavity to communicate freely with the oral cavity without the need for an additional drainage device. Decompression, in contrast, involves the insertion of a tube, drain, or appliance‐retained conduit into the cystic cavity through a small surgical opening, without suturing the cystic lining to the oral mucosa; this maintains a controlled and continuous outflow that gradually reduces intracystic pressure, whereas the size of the opening can be adjusted as the lesion resolves. In the present series, appliance‐retained drains were used to establish and maintain this controlled communication; therefore, the term decompression, rather than marsupialization, is used consistently throughout this report to describe the technique performed in all three cases [14].

Despite the growing use of appliance‐supported decompression in the management of odontogenic cysts, the existing literature on primary tooth‐associated lesions remains largely limited to isolated case reports, and comparative data regarding different appliance designs and their clinical performance are scarce [13, 15]. Furthermore, most previous reports have focused on single‐case outcomes, and appliance selection in relation to lesion size, location, and the degree of permanent tooth displacement has not been widely discussed, leaving a relative gap in the available evidence for clinicians managing these lesions in the mixed dentition period. Unlike conventional marsupialization techniques that rely on temporary obturators or gauze packing requiring frequent replacement and carrying a risk of premature closure, appliance‐supported decompression systems provide continuous, self‐retentive drainage while simultaneously acting as space maintainers, thereby offering a more predictable and less operator‐dependent alternative. By presenting a series of cases managed with individualized fixed and removable appliances and relating appliance design to lesion characteristics and treatment outcomes, the present study aims to contribute additional clinical insight to this relatively underreported area in the pediatric population.

This study is aimed at presenting a series of pediatric radicular cyst cases, the most frequently encountered inflammatory odontogenic cyst of the jaws in children, successfully treated with conservative appliance‐supported decompression and at discussing the clinical rationale, comparative advantages, and limitations of the different decompression modalities employed.

## 2. Case Presentations

### 2.1. Case 1

#### 2.1.1. Patient Information

An 8‐year‐old male patient presented to the Department of Pediatric Dentistry with a complaint of painful swelling in the left mandibular region. There was no associated fever or numbness of the cheek or lip. The patient had no known comorbidities and appeared generally healthy for his age. His medical history revealed no systemic diseases or previous traumatic injuries in the affected area. The mucosa overlying the swelling was adherent to the lesion. The patient was classified as having a severe caries risk.

Initial radiographs were obtained on 30 December 2022, to aid in formulating the treatment plan. The teeth present in the affected region included a primary mandibular left molar exhibiting physiologic mobility prior to exfoliation, a second primary molar that had previously undergone pulpotomy, and pit and fissure caries associated with the permanent mandibular left first molar.

#### 2.1.2. Clinical Findings

Extraoral examination revealed a diffuse swelling in the left lower third of the face, measuring approximately 2 × 3 cm, extending anteroposteriorly from the left corner of the mouth to the left angle of the mandible, and superoinferiorly from 1 cm below the left alatragal line to the lower border of the left mandible. The swelling was firm in consistency and tender to palpation. Intraoral soft tissue examination revealed a diffuse swelling of the left buccal mucosa, measuring approximately 2 × 3 cm, extending from the distal aspect of tooth 32 to the distal aspect of tooth 36 and obliterating the buccal vestibule. On palpation, cortical expansion of the buccal cortex was noted, and the swelling was firm in consistency. Percussion sensitivity was noted in teeth 74, 75, and 36.

#### 2.1.3. Diagnostic Assessment

Radiographic examination revealed a significant radiolucent lesion extending from the region of tooth 32 to the mandibular angle on the left side. Displacement of the succedaneous permanent premolars and canine was also observed (Figure 1). Given the extent of the lesion on panoramic radiography, CBCT was additionally obtained for three‐dimensional evaluation. CBCT of the mandible revealed a well‐defined, monolocular hypodense lesion measuring approximately 20 × 20 × 58 mm, extending from the distal aspect of tooth 32 posteriorly to the mandibular ramus, and inferiorly to the base of the mandible. The tooth 33 and tooth 32 were located anterior to the lesion, whereas tooth 36 and the tooth 37 were adjacent to its posterior border. The roots of tooth 75 and the crowns of the teeth 34 and 35 were located within the boundaries of the lesion. Destruction and expansion of both the buccal and lingual cortical plates were noted. The lesion was observed to displace the mandibular canal buccally, and the mental foramen could not be clearly identified. Differential diagnoses included a radicular cyst, dentigerous cyst, and odontogenic keratocyst (Figure 2).

**Figure 1:**
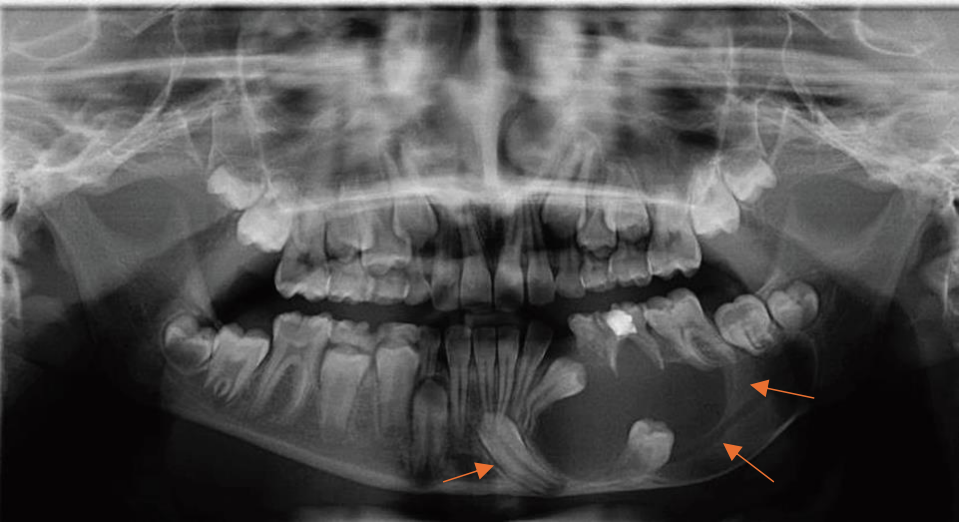
Panoramic radiograph obtained at the initial evaluation appointment, demonstrating the extent of bony destruction associated with the lower left mandibular lesion.

**Figure 2:**
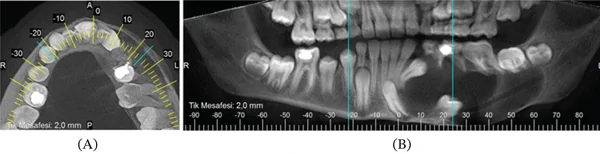
CBCT images of the mandibular lesion in Case 1. (A) Axial view showing the buccolingual extent of the lesion. (B) Cross‐sectional reconstruction showing its anteroposterior extent, with cyan lines marking the mesial and distal borders. R, right; L, left.

#### 2.1.4. Therapeutic Intervention

Prior to the procedure, written informed consent was obtained from the patient′s parent, and the patient presented to the dental clinic on 5 January 2023 for the decompression procedure. Local anesthesia was achieved in the lower left quadrant using 2% lidocaine with 1:100,000 epinephrine (0.017 mg) administered via a mandibular block, supplemented by 4% articaine with 1:100,000 epinephrine (0.017 mg) via local infiltration. Primary teeth 74 and 75 in the lower left quadrant were extracted, after which the cystic lining was identified and surgically opened (Figure 3). The excised tissue specimen was sent to the pathology laboratory for histopathological evaluation.

**Figure 3:**
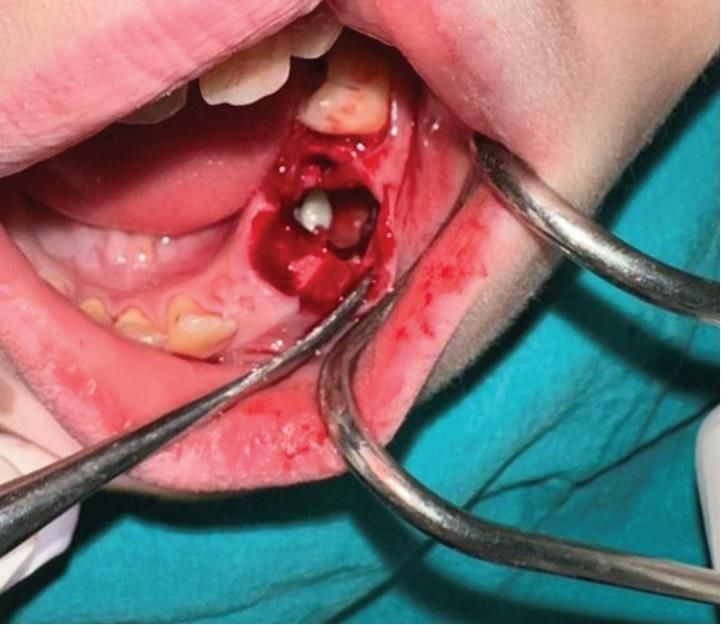
Lower left vestibule after extraction of teeth 74 and 75.

Microscopic evaluation of the excised specimen demonstrated a cyst wall composed of epithelial tissue overlying a fibrous connective tissue stroma, with dense infiltration of chronic inflammatory cells throughout the underlying tissue. These histopathological features were compatible with a radicular cyst.

Given the extensive size of the lesion and its proximity to the developing succedaneous premolars and canine, a fixed appliance was preferred to ensure stable and uninterrupted space maintenance throughout the prolonged decompression period. Considering the patient′s young age, a fixed design was also selected to eliminate reliance on patient compliance for appliance wear, thereby reducing the risk of drain displacement or appliance loss that could compromise the decompression process. The lingual arch configuration further allowed the drain to be securely integrated into the appliance itself, minimizing soft‐tissue irritation while maintaining continuous drainage.

A tooth‐borne lingual arch appliance was fabricated to preserve the surgical space until eruption of the permanent teeth. A silicone impression was obtained prior to the surgical procedure, allowing fabrication of the appliance in advance. The appliance was designed to be supported by the mandibular permanent molars and constructed in a lingual arch configuration. In the region corresponding to the cystic cavity, a customized loop was incorporated into the lingual arch, and a drain was positioned within this loop. The lingual arch was fabricated from 0.040‐inch (1.0 mm) round stainless steel wire, a gauge slightly heavier than the 0.036‐inch wire conventionally used for lingual holding arches. This increased rigidity was selected to withstand the additional structural demand imposed by the customized loop incorporated for drain retention, reducing the risk of wire distortion or fracture during the prolonged decompression period. The appliance was retained by stainless steel bands cemented to the mandibular first permanent molars bilaterally (Figure 4). The appliance was placed intraoperatively at the time of surgery.

**Figure 4:**
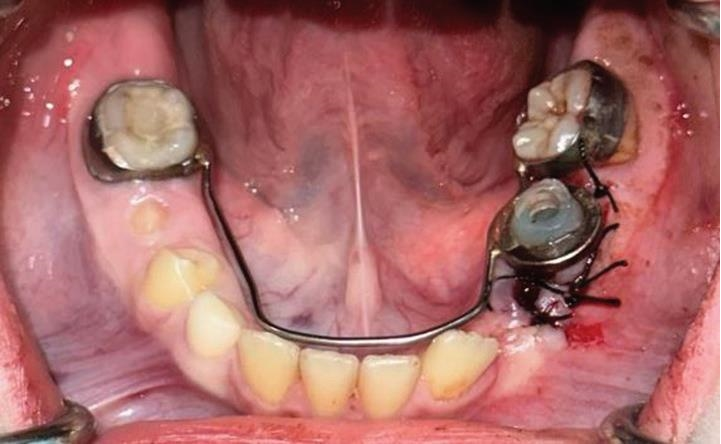
Intraoral view of the appliance immediately following placement on the day of extraction.

By integrating the drain into the appliance rather than relying on soft‐tissue support, the drain was maintained in a stable and secure position, significantly reducing the risk of displacement. This design eliminated common complications associated with tissue‐supported drains, such as mucosal irritation and patient discomfort. In addition, the tooth‐borne nature of the appliance ensured maintenance of drainage patency throughout the healing period.

The parents were given detailed postoperative guidance, covering oral hygiene maintenance, dietary modification (soft diet), and awareness of potential infection signs. Analgesics and antiseptic mouthwash were prescribed on an as‐needed basis. In addition, caregivers were instructed to irrigate the cystic cavity through the drain loop twice daily using physiological saline solution delivered with a blunt‐tipped syringe, followed by gentle aspiration of the irrigant and any accumulated exudate. Detailed hands‐on training was provided to the parent at the time of appliance placement, including proper syringe positioning to avoid trauma to the surrounding mucosa. The parents were also instructed to monitor for signs of appliance loosening, band fracture, or drain displacement and to contact the clinic promptly if any of these occurred.

The loop design also allowed easy access for caregiver‐assisted aspiration, facilitating home management of cystic fluid and reducing the need for frequent clinical follow up visits. Overall, this appliance provided effective space maintenance, improved patient comfort, and enhanced clinical control during the decompression phase.

#### 2.1.5. Follow‐Up and Outcomes

As the cyst progressively decreased in size and the associated teeth continued to erupt, the length of the drain was gradually reduced over time to accommodate healing and eruption dynamics. During the follow‐up period, 7 months after the initial presentation, fracture of the band of the space‐maintaining appliance was noted, necessitating replacement of the appliance. At 20 months following the initial presentation, a follow‐up panoramic radiograph was obtained for evaluation purposes (Figure 5). Complete eruption of both premolars in the lower left quadrant was observed, along with adequate bone fill of the previous defect, satisfactory bone levels, and no evidence of periodontal pathology. However, due to arch‐length deficiency, the mandibular left canine (tooth 33) remained impacted. A decision was made to manage this finding with continued clinical and radiographic follow‐up. No clinical or radiographic evidence of recurrence was observed throughout the follow‐up period, and the patient remained asymptomatic at the final evaluation.

**Figure 5:**
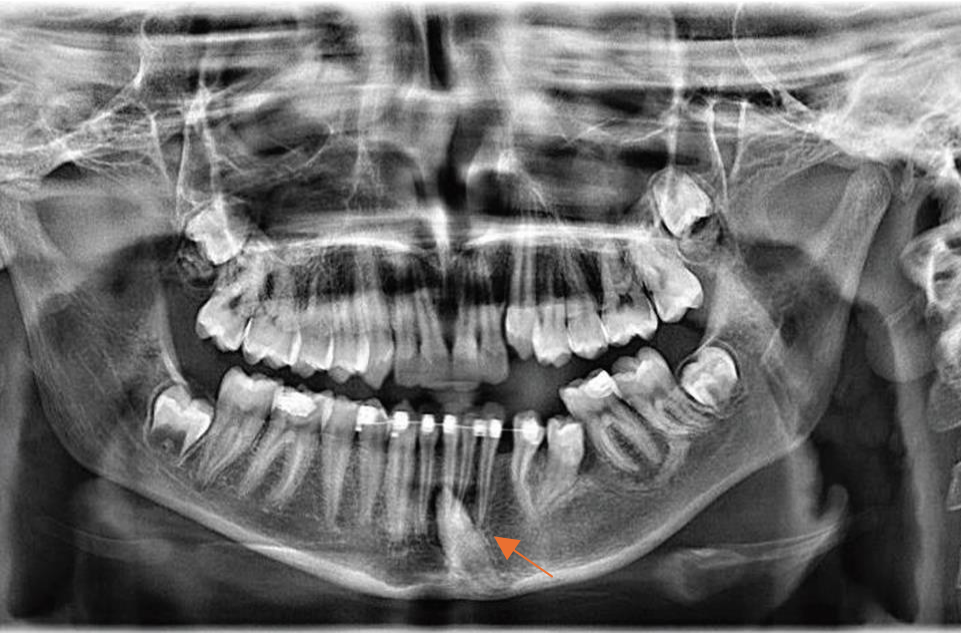
A panoramic radiograph 20 months after operation showing complete bony fill with no residual cyst in the lower left quadrant.

### 2.2. Case 2

#### 2.2.1. Patient Information

A 7‐year‐old male patient presented to the Department of Pediatric Dentistry with the chief complaint of pain in the right posterior region of the mandible. The patient was in good general health, with no history of systemic disease, regular medication use, or known allergies. There was no history of previous traumatic injury in the affected region. The dental history revealed previous pulpotomy treatment of the mandibular right second primary molar (tooth 85), which had subsequently been restored. An active carious lesion was present on the mandibular right first primary molar (tooth 84). Oral hygiene was considered poor, and the patient demonstrated a high caries experience.

#### 2.2.2. Clinical Findings

Extraoral examination revealed no facial asymmetry or clinically evident swelling. Intraoral examination showed an active carious lesion involving the mandibular right first primary molar (tooth 84) and a previously restored mandibular right second primary molar (tooth 85). Buccal cortical expansion was evident in the right posterior mandibular region. Mobility and sensitivity to percussion were noted in the affected primary molars. The patient exhibited limited cooperation during the clinical examination and showed a pronounced response to palpation of the affected region, suggesting local tenderness and discomfort. No signs of acute soft‐tissue infection were observed.

#### 2.2.3. Diagnostic Assessment

Radiographic evaluation, including panoramic and periapical radiographs, demonstrated a well‐defined, unilocular radiolucent lesion measuring approximately 35 × 25 mm (mesiodistal × superoinferior) on the panoramic radiograph, associated with the roots of the mandibular right primary molars (teeth 84 and 85). The lesion was closely associated with the developing permanent successors and caused displacement of the underlying premolar tooth germ from its expected position (Figure 6). Based on the clinical and radiographic findings, together with the history of previous pulp therapy involving tooth 85, a radicular cyst was considered the primary provisional diagnosis. Differential diagnoses included a dentigerous cyst and an odontogenic keratocyst. The patient was subsequently referred to the Department of Oral and Maxillofacial Surgery for further evaluation and management.

**Figure 6:**
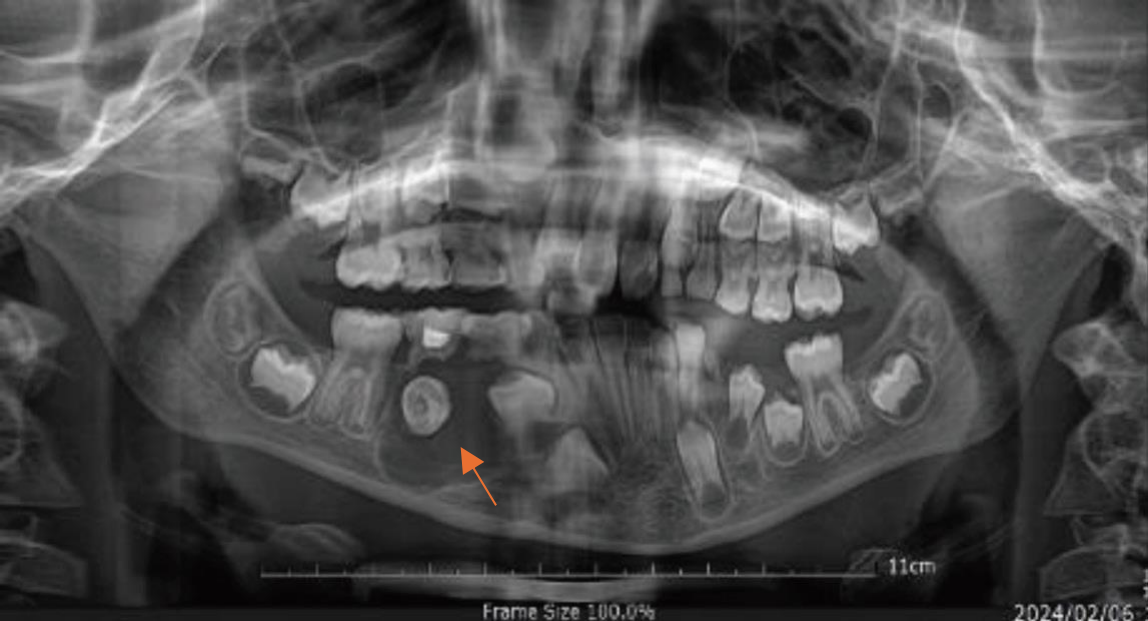
Preoperative panoramic radiograph (Case 2).

#### 2.2.4. Therapeutic Intervention

Following comprehensive evaluation, a conservative surgical approach was planned to preserve the underlying permanent tooth germs. Under local anesthesia, teeth 84 and 85 were atraumatically extracted, and surgical access to the cystic cavity was obtained. A drain was inserted into the cyst cavity to achieve decompression and was stabilized to allow continuous drainage into the oral cavity (Figure 7). The excised cystic lining and associated soft tissue were submitted for histopathologic examination. Postoperative instructions were provided to the patient and parents, including antibiotic therapy, analgesic medication, and oral hygiene measures.

**Figure 7:**
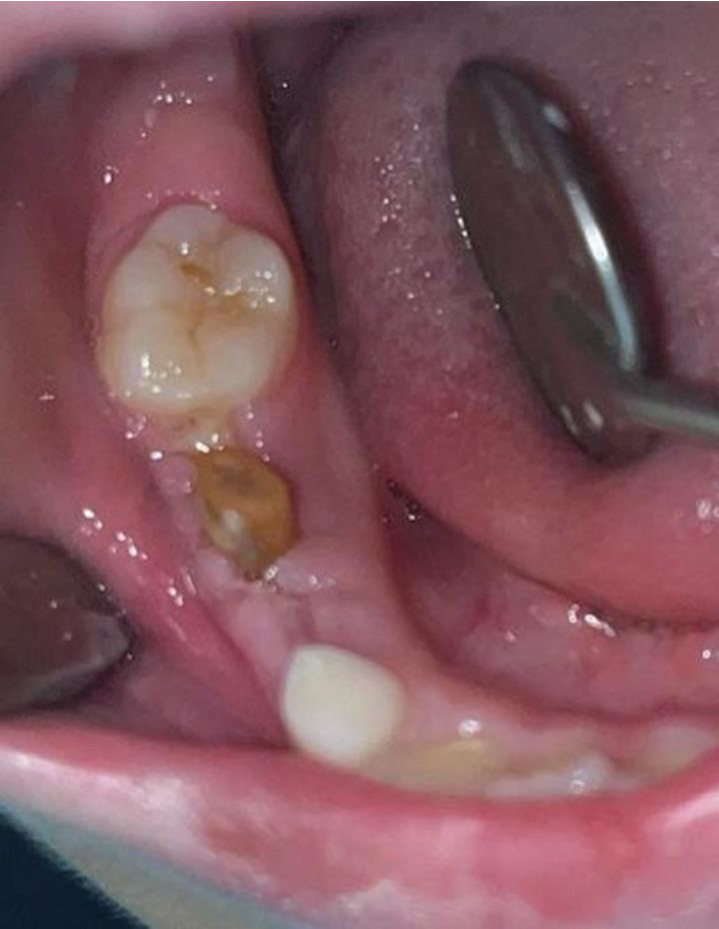
Intraoral photograph showing fixation of the drain after extraction of the primary teeth.

Following initial soft‐tissue healing, a custom‐made fixed, tooth‐borne decompression appliance was fabricated. The appliance consisted of a lingual arch–type framework constructed from 0.040‐inch (1.0 mm) round stainless‐steel wire and anchored to the posterior teeth. A customized extension was incorporated into the right mandibular region to stabilize the decompression drain and maintain patency of the cystic opening. In addition to providing stable and continuous decompression, the tooth‐borne design maintained the space created following extraction of teeth 84 and 85.

Histopathological analysis of the submitted specimens revealed a cystic lesion lined by nonkeratinized stratified squamous epithelium with a fibrous connective tissue wall exhibiting chronic inflammatory cell infiltration. These findings were consistent with a diagnosis of a radicular cyst.

Fifteen days after drain placement, the patient was recalled to the Department of Pediatric Dentistry for further management. Clinical examination showed satisfactory healing of the surrounding soft tissues, with no signs of infection or inflammation. At this appointment, the drain was temporarily removed, and an impression of the mandibular arch was obtained using a silicone‐based impression material. Immediately after impression taking, the drain was reinserted into the cyst cavity to maintain decompression.

At the request of the patient′s parents, a fixed decompression appliance was fabricated and subsequently cemented intraorally (Figure 8). The appliance was designed to stabilize the drain and maintain continuous decompression of the cystic cavity. Given the patient′s limited cooperation, the fixed design was considered particularly advantageous because it ensured uninterrupted appliance wear without relying on patient compliance, thereby minimizing the risk of appliance loss, inadvertent removal, or disruption of the decompression process associated with removable systems. Due to the fixed nature of the appliance, the patient was instructed to perform daily irrigation of the drain using physiological saline solution, as removal of the appliance was not possible. Detailed oral hygiene and irrigation instructions were provided to both the patient and the parents.

**Figure 8:**
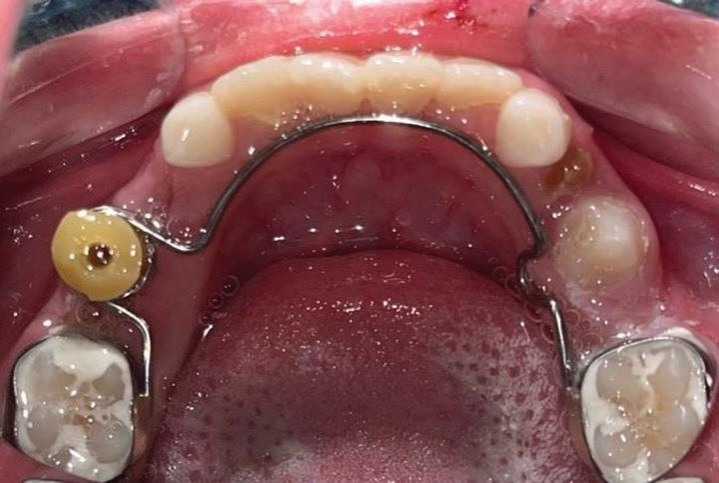
Intraoral view of the appliance.

#### 2.2.5. Follow‐Up and Outcomes

The patient was followed at 1‐month intervals, during which clinical and radiographic evaluations were performed. Progressive reduction in the size of the cystic cavity was observed, along with signs of bone regeneration in the affected area. No complications, such as infection, appliance failure, or patient discomfort, were encountered during the follow‐up period.

At the 3‐month follow‐up, no measurable residual radiolucent lesion was evident radiographically, indicating complete radiographic resolution of the cystic cavity (Figure 9). The associated permanent successors showed favorable eruption and continued development without the need for orthodontic intervention. No adverse effects on tooth development, eruption pattern, or the surrounding periodontal tissues were observed. The patient remained asymptomatic, with no clinical or radiographic evidence of recurrence at the final follow‐up.

**Figure 9:**
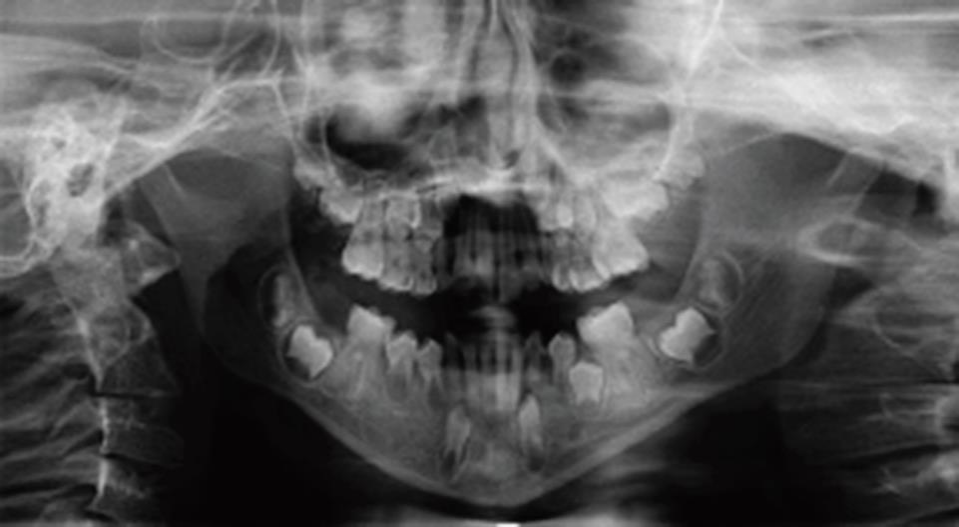
A panoramic radiograph at 3‐month follow‐up.

### 2.3. Case 3

#### 2.3.1. Patient Information

A 7‐year‐old male patient presented to the Department of Pediatric Dentistry for evaluation and treatment of dental caries affecting the primary molars in the right posterior region of the mandible. The patient was in good general health, with no history of systemic disease, regular medication use, or known allergies. There was no history of previous traumatic injury in the affected region. The mandibular right primary molars (teeth 84 and 85) had no history of previous restorative or pulp therapy. Both teeth exhibited extensive deep carious lesions with substantial coronal destruction. The patient reported no spontaneous pain, and there was no history of abscess formation or swelling.

#### 2.3.2. Clinical Findings

Extraoral examination revealed no facial asymmetry or clinically evident swelling. Intraoral examination demonstrated extensive deep carious lesions with substantial coronal destruction involving the mandibular right primary molars (teeth 84 and 85). No intraoral swelling, abscess formation, or other signs of acute soft‐tissue infection were observed. Both affected primary molars exhibited sensitivity to percussion.

#### 2.3.3. Diagnostic Assessment

Radiographic evaluation, including panoramic and periapical radiographs, revealed a well‐defined, unilocular radiolucent lesion measuring approximately 30 × 20 mm (mesiodistal × superoinferior) on the panoramic radiograph, associated with the roots of the mandibular right primary molars (teeth 84 and 85) (Figure 10). The lesion was closely associated with the developing permanent successors. Considering the radiographic characteristics, lesion size, and the extensive deep carious lesions involving the affected primary teeth, a radicular cyst was considered the primary provisional diagnosis. Differential diagnoses included a dentigerous cyst and an odontogenic keratocyst. Due to the lesion′s size, anatomical location, and its potential impact on the underlying permanent tooth germ, the patient was referred to the Department of Oral and Maxillofacial Surgery for further evaluation.

**Figure 10:**
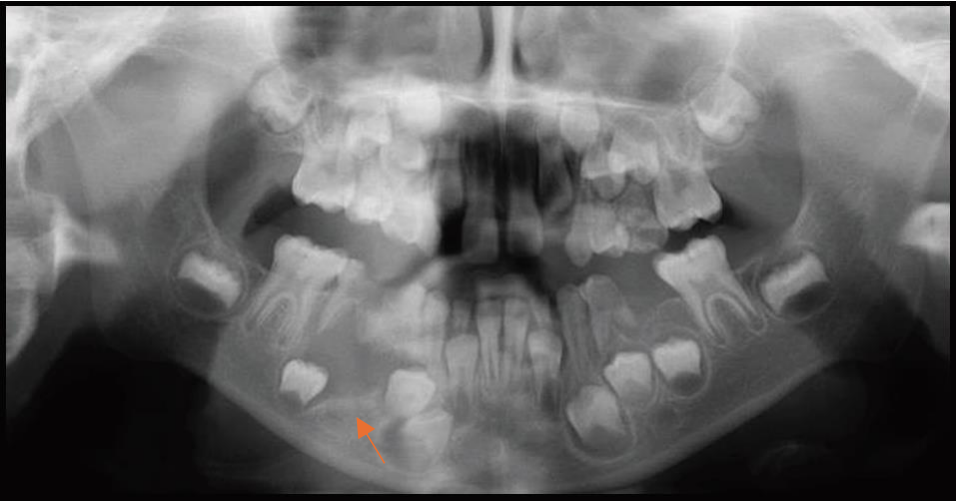
Preoperative panoramic radiograph (Case 3).

#### 2.3.4. Therapeutic Intervention

Following initial healing, a custom‐made removable decompression appliance was fabricated. The appliance consisted of an acrylic resin base incorporating stainless‐steel wire retentive components for stabilization on the remaining teeth. A tubular acrylic extension was incorporated into the appliance in the region corresponding to the cystic cavity and extended into the decompression opening to maintain patency and continuous communication between the cystic cavity and the oral environment. The removable design allowed the appliance to be taken out for cleaning and facilitated access to the surgical site for irrigation and oral hygiene.

In contrast to Case 2, in which a fixed appliance was preferred because of the patient′s limited cooperation and the need to ensure uninterrupted decompression without relying on appliance wear compliance, a removable appliance was considered appropriate in the present case because the lesion was smaller and well circumscribed and the patient was sufficiently cooperative to comply with appliance use. The removable design also provided easier access to the decompression site for daily irrigation and oral hygiene, while allowing adequate stabilization of the drain. Thus, appliance selection was individualized according to lesion characteristics, anticipated patient compliance, and hygiene requirements.

The excised tissue was forwarded for histopathological evaluation, which demonstrated a cystic cavity lined by nonkeratinized epithelium of variable thickness, exhibiting an arcading pattern of epithelial proliferation. The underlying connective tissue showed dense infiltration by chronic inflammatory cells.

Fifteen days after drain placement, the patient was referred back to the Department of Pediatric Dentistry. Clinical examination revealed healthy surrounding soft tissues with no signs of infection. At this stage, while maintaining the drain in position, an impression of the mandibular arch was obtained using a silicone‐based impression material. Following impression taking, the drain was reinserted and stabilized in the cyst cavity. Based on the obtained impression, a removable decompression appliance was fabricated to facilitate controlled reduction of the cystic cavity and to improve oral hygiene maintenance.

Approximately 1 week after impression taking, the custom‐made removable decompression appliance was delivered to the patient (Figure 11). Detailed instructions regarding appliance use were provided to both the patient and the parents. The patient was advised to wear the appliance continuously throughout the day and to remove it only during meals. In addition, the patient was instructed to irrigate the drain and cyst cavity daily using physiological saline solution.

**Figure 11:**
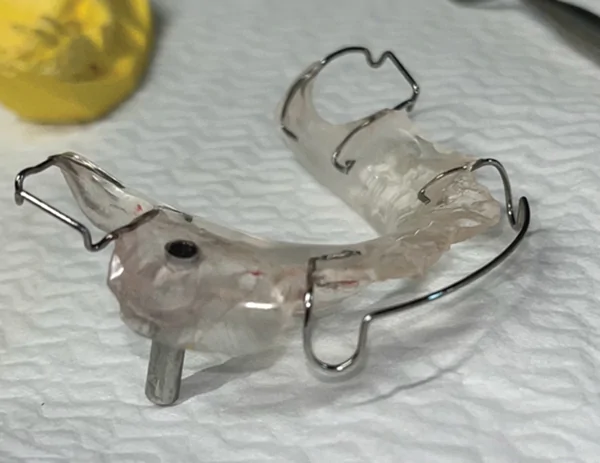
Removable decompression appliance.

#### 2.3.5. Follow‐Up and Outcomes

During follow‐up visits, the patient demonstrated good compliance with appliance use and irrigation protocols. Serial radiographic evaluations demonstrated a progressive reduction in the cystic cavity from its initial dimensions of approximately 30 × 20 mm, accompanied by progressive bone regeneration in the affected area. At the 15‐month follow‐up, a marked reduction in the radiolucent lesion was observed; however, complete radiographic resolution had not yet been achieved (Figure 12). No adverse effects on the developing permanent tooth germs were observed, and no appliance failure or other treatment‐related complications occurred during the follow‐up period. As radiographic healing was still ongoing at the final evaluation, definitive assessment of recurrence was not yet possible.

**Figure 12:**
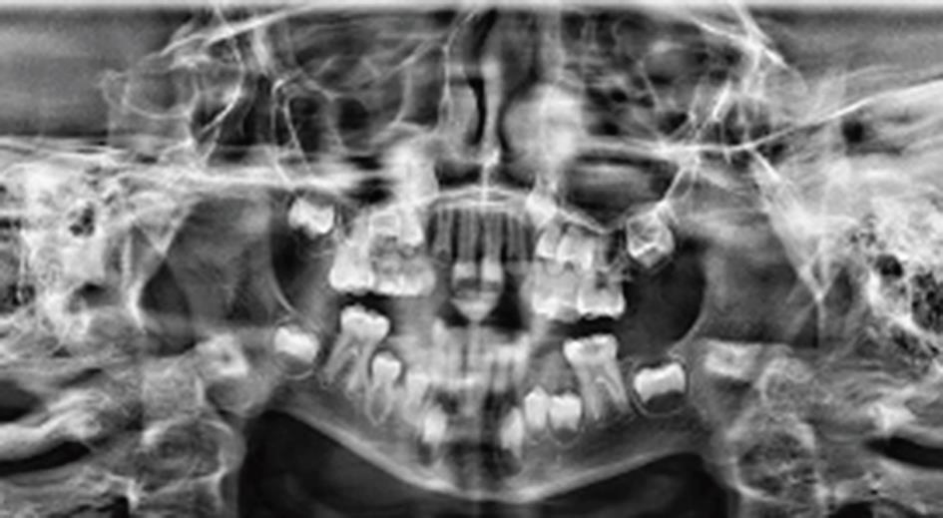
A panoramic radiograph 15 months after operation.

Treatment and follow‐up characteristics of the three cases are summarized in Tables 1 and 2.

**Table 1:** Treatment and follow‐up timeline for the three cases.

Case	Initial diagnosis	Extraction	Drain placement	Appliance insertion	Follow‐up intervals	Radiographic healing	Eruption outcome
1	30 Dec 2022	5 Jan 2023	5 Jan 2023 (intraoperative)	5 Jan 2023 (intraoperative)	Monthly/band fracture at 7 months	Complete bony fill at 20 months	Complete eruption of premolars; canine impacted
2	02 Feb 2024	16 Feb 2024	Intraoperative	15 days after drain placement	Monthly	Complete resolution at 3 months	Spontaneous eruption
3	25 Aug 2023	08 Sep 2023	Intraoperative	~1 week after impression (impression taken 15 days after drain placement)	Monthly	Ongoing at 15 months (not yet complete)	Not yet completed/under follow‐up

**Table 2:** Comparative summary of the three cases.

Case	Age/Sex	Teeth	Chief complaint	Lesion size (mm)	Histopathology	Appliance	Decompression duration	Follow‐up	Outcome	Complication
1	8y/M	74, 75	Painful swelling	20 × 20 × 58 mm	Cyst wall with epithelium over fibrous connective tissue, chronic inflammatory infiltrate; compatible with radicular cyst	Fixed (tooth‐borne lingual arch)	16 months	20 months	Complete bony fill; premolars erupted; canine impacted	Band fracture at 7 months, appliance replaced
2	7y/M	84, 85	Pain, posterior mandible	35 × 25 mm	Nonkeratinized stratified squamous epithelium, chronic inflammatory infiltrate; consistent with radicular cyst	Fixed (cemented)	3 months	3 months	Complete resolution; spontaneous eruption	None reported
3	7y/M	84, 85	Caries, posterior mandible	30 × 20 mm	Nonkeratinized epithelium, variable thickness, arcading pattern; dense chronic inflammatory infiltrate	Removable	15 months	15 months	Gradual reduction; bone regeneration ongoing	None reported

## 3. Discussion

Radicular cysts occur with relatively low frequency in the pediatric population; however, they represent a significant therapeutic challenge for clinicians due to the need to preserve the underlying permanent tooth germ. Odontogenic cysts are generally classified as developmental or inflammatory, with radicular cysts being the most common inflammatory odontogenic cyst arising secondary to pulp necrosis and chronic periradicular inflammation [15]. Historically, enucleation combined with extraction of the associated tooth has long been considered the gold standard treatment. In recent years, however, accumulating evidence has demonstrated that conservative treatment approaches, particularly decompression and marsupialization, are increasingly favored in pediatric patients [16]. Pei et al. [17], in a retrospective study, clearly demonstrated that decompression is an effective treatment modality for radicular cysts associated with primary teeth, despite their low incidence. Most contemporary reports describe spontaneous eruption of permanent successors with no recurrence, emphasizing the importance of conservative management during periods of active craniofacial growth [17].

Pulp necrosis due to dental caries is widely accepted as the principal etiological factor in the development of radicular cysts, as observed in Case 2 of the present series [3]. Radicular cysts are typically asymptomatic and are often discovered incidentally during routine radiographic examination [4]. As demonstrated in Case 1, periradicular inflammation may persist or even progress after pulp therapy in primary teeth and remain clinically silent for extended periods, highlighting the necessity of long‐term follow‐up of pulpotomized or pulpectomized deciduous teeth. Several authors have suggested that rapid cyst enlargement may be related to intracanal medicaments used during pulp therapy, acting as continuous antigenic stimuli [18, 19]. Grundy et al. [19] reported that antigenic necrotic materials produced by pulpal therapeutic agents—particularly those containing phenol compounds—may contribute to sustained periradicular inflammation and cyst formation. Importantly, this does not contraindicate pulp therapy in primary teeth, given the overall low incidence of radicular cysts, but rather underscores the importance of periodic monitoring.

In the present case report, all cysts were located in the mandible and were associated with primary molars. This finding is consistent with previous studies, including Manekar et al., [20] which reported that mandibular primary molars are the teeth most frequently involved in radicular cysts. The predilection for mandibular primary molars has been attributed to their greater susceptibility to caries and pulp involvement [19, 20]. Specific clinical features have been described for radicular cysts associated with pulpotomized primary molars, including large lesion size, buccal cortical expansion, and rapid growth, findings that were partially observed in the present cases.

Accurate diagnosis of radicular cysts requires careful correlation of patient history, clinical examination, radiographic findings, and histopathological evaluation. Radiographically, these lesions typically appear as well‐defined periradicular radiolucencies with a thin reactive cortical border and may be associated with buccal plate expansion and displacement of developing permanent tooth buds [21].

Marsupialization and decompression, as defined above, are both widely accepted conservative approaches for the management of large odontogenic cysts, and the choice between them is generally guided by lesion size, anatomical extent, and proximity to vital structures such as the mandibular canal and permanent tooth germs, with the shared goals of reducing surgical morbidity, avoiding excessive bone loss, minimizing growth disturbances, and reducing the risk of pathological fracture. Decompression, as applied in the present series, reduces intracystic pressure, allowing gradual bone regeneration and repositioning of displaced teeth [22]. de Molon et al. proposed that pressure reduction through decompression facilitates restoration of normal anatomy by surrounding bone and periosteum [23]. More recently, Ben Arbia et al. [24] similarly reported effective resolution of large inflammatory dentigerous cysts in children managed with marsupialization, reinforcing the broader applicability of conservative, minimally invasive techniques across different types of inflammatory odontogenic cysts in the growing jaw.

The three cases in this series illustrate how appliance design can be individualized according to lesion characteristics, anticipated treatment duration, and expected patient and parental compliance, and each design carried distinct advantages and limitations. In Case 1, a tooth‐borne fixed lingual arch appliance with a loop‐integrated drain was selected because of the extensive size of the lesion and the need for long‐term space maintenance until eruption of two displaced premolars. Anchoring the drain to the appliance rather than to soft tissue kept it in a stable position and minimized common complications reported with soft tissue–supported drains, such as mucosal irritation or displacement, while allowing caregiver‐assisted aspiration at home and reducing the need for frequent clinical visits [13]. This design nonetheless carries an inherent mechanical risk, as evidenced by the band fracture that required appliance replacement during follow‐up, indicating that fixed lingual arch appliances require periodic monitoring for structural integrity over extended treatment periods. In Case 2, a fixed appliance cemented directly onto the dentition was chosen, at the family′s request, to stabilize the drain and eliminate the risk of appliance loss or noncompliance inherent to removable systems. Its principal limitation was that, because the appliance could not be removed by the patient, daily saline irrigation and oral hygiene around the drain depended entirely on patient and parental cooperation.

In Case 3, a removable decompression appliance was preferred because the lesion was smaller and well‐circumscribed, and removability was judged to offer a hygienic advantage over a fixed design for a lesion of this extent. This design allowed the patient to remove the appliance for hygiene and meals; the resin extension incorporated into the appliance allowed effective decompression, prevented food debris accumulation, and reduced fibrous tissue formation that could interfere with eruption of the permanent successor. Its effectiveness was, however, more directly dependent on patient adherence to near‐continuous wear and daily irrigation, since inconsistent use could compromise decompression; as emphasized in the literature, frequent irrigation of the cystic cavity is essential, making patient and parental compliance a critical determinant of success [25]. Taken together, these observations suggest that fixed, tooth‐borne appliances may be more appropriate for larger lesions requiring prolonged decompression and reliable long‐term space maintenance, whereas removable appliances may be better suited to smaller, well‐defined lesions in cooperative patients for whom hygiene access is a priority; however, in the absence of standardized selection criteria, this rationale remains based on clinical judgment rather than on a validated treatment algorithm.

Patient age plays a crucial role in predicting spontaneous eruption following conservative treatment. Systematic reviews have shown significantly higher eruption rates in children younger than 10 years [16]. Pei et al. also reported that decompression time in patients younger than 18 years was significantly shorter than in adults, reflecting higher osteogenic potential in children [17]. All patients in the present series were under 10 years of age, which likely contributed to the favorable outcomes observed. Previous studies, including those by Berti et al., have reported spontaneous alignment and eruption of permanent teeth even when initial tooth germ positions were severely displaced [26].

Although spontaneous eruption or favorable repositioning of the permanent successors was achieved in all three patients of this series, these favorable outcomes should be interpreted with caution given the small sample size and should not be generalized to all cases of primary tooth radicular cysts. Spontaneous eruption following decompression is not a universal outcome and depends on the interplay of several factors, including patient age, initial lesion size, the degree of tooth germ displacement, the stage of root development of the permanent successor at presentation, and the amount of eruption space available within the dental arch, as illustrated by the impaction of the mandibular left canine (tooth 33) despite otherwise successful bony healing in Case 1, attributable to arch‐length deficiency rather than to treatment failure. Furthermore, previous literature has reported that a substantial proportion of permanent successors may exhibit root development disturbances following cyst resolution, including ectopic eruption, incomplete root formation, dilaceration, and wide root canals [16]. Therefore, long‐term follow‐up of the developing permanent dentition is essential, and early orthodontic or restorative intervention may be required to prolong the survival of affected teeth.

Despite the encouraging outcomes associated with decompression, the existing literature is limited predominantly to retrospective studies and case reports, a limitation shared by the present report. Several additional limitations should be acknowledged. First, the small number of cases and the absence of a comparison or control group limit the generalizability of the findings and preclude any direct, statistically supported comparison of the fixed and removable decompression modalities described. Second, appliance selection and treatment planning were individualized based on lesion characteristics and clinician judgment rather than on standardized, evidence‐based criteria, since no validated protocol currently exists to guide the choice between fixed and removable decompression appliances in primary tooth radicular cysts. Third, reduction in lesion size and bone regeneration were assessed qualitatively on sequential two‐dimensional radiographs rather than through quantitative volumetric or linear measurements, which may have introduced observer‐dependent variability in the interpretation of healing. Finally, as is inherent to any case series, the retrospective, nonrandomized nature of the follow‐up and the absence of a control group preclude causal conclusions regarding the relative efficacy of the treatment modalities described. These findings collectively highlight the need for larger prospective, and ideally comparative or randomized, studies to establish standardized, evidence‐based treatment protocols for radicular cysts associated with primary teeth.

## 4. Conclusion

Inflammatory odontogenic cysts associated with primary teeth may progress silently in pediatric patients, yet result in extensive bone destruction and significant displacement of developing permanent tooth germs. This case report demonstrates that conservative management consisting of extraction of the affected primary teeth combined with appliance‐supported decompression is an effective and minimally invasive treatment approach. Such management not only facilitates cyst size reduction and bone regeneration but also preserves the eruption potential and developmental integrity of permanent successors. Individualized selection of fixed or removable decompression appliances, based on lesion characteristics and patient compliance, plays a crucial role in achieving successful outcomes. Even in cases with severe initial displacement, careful decompression and long‐term follow‐up may allow spontaneous eruption and favorable positioning of permanent teeth, thereby reducing the need for aggressive surgical interventions in growing patients.

## Author Contributions

Erenay Alpayçetin: conceptualization, data curation, formal analysis, investigation, methodology, visualization, writing – original draft, and writing – review and editing. Merve Mısır: data curation and writing – original draft. Elif Bahar Tuna İnce: investigation, supervision and validation, and writing – original draft. Mine Koruyucu: investigation, methodology, writing – review and editing, supervision and validation. Koray Gençay: conceptualization, investigation, methodology, supervision and validation.

## Funding

No funding was received for this manuscript.

## Ethics Statement

Ethics approval was not required for this case report, as it describes three patients and does not involve experimental interventions. Written informed consent for participation was obtained from the patients′ legal guardians.

## Consent

Written informed consent was obtained from the patients parents for publication of this case report and the accompanying clinical images. A copy of the consent is available for review by the Editor of this journal.

## Conflicts of Interest

The authors declare no conflicts interests.

## Data Availability

The data supporting the findings of this case report (including clinical images and radiographs) are available from the corresponding author upon reasonable request. All patient‐identifiable information has been removed to ensure confidentiality.
